# Expression Profiling of Exosomal miRNAs Derived from Human Esophageal Cancer Cells by Solexa High-Throughput Sequencing

**DOI:** 10.3390/ijms150915530

**Published:** 2014-09-02

**Authors:** Juan Liao, Ran Liu, Lihong Yin, Yuepu Pu

**Affiliations:** Key Laboratory of Environmental Medicine Engineering, Ministry of Education, School of Public Health, Southeast University, Nanjing 210009, China; E-Mails: liaojuan1128@gmail.com (J.L.); lhyin@seu.edu.cn (L.Y.); yppu@seu.edu.cn (Y.P.)

**Keywords:** esophageal cancer, exosome, microRNA, expression profile

## Abstract

Cellular genetic materials, such as microRNAs (miRNAs), mRNAs and proteins, are packaged inside exosomes, small membrane vesicles of endocytic origin that are released into the extracellular environment. These cellular genetic materials can be delivered into recipient cells, where they exert their respective biological effects. However, the miRNA profiles and biological functions of exosomes secreted by cancer cells remain unknown. The present study explored the miRNA expression profile and distribution characteristics of exosomes derived from human esophageal cancer cells through Solexa high-throughput sequencing. Results showed that 56,421 (2.94%) unique sequences in cells and 7727 (0.63%) in exosomes matched known miRNAs. A total of 342 and 48 known miRNAs were identified in cells and exosomes, respectively. Moreover, 64 and 32 novel miRNAs were predicted in cells and exosomes, respectively. Significant differences in miRNA expression profiles were found between human esophageal cancer cells and exosomes. These findings provided new insights into the characteristics of miRNAs in exosomes derived from human esophageal cancer cells and the specific roles of miRNAs in intercellular communication mediated by exosomes in esophageal cancer.

## 1. Introduction

Exosomes are small (40–100 nm) membrane vesicles that are derived from the invagination of endosomal compartments called multivesicular bodies and then released into the extracellular environment by most cell types [1]. Recent studies have reported that exosomes exist in the culture supernatant of numerous eukaryotic cells, including cytotoxic T-lymphocytes [2], B-lymphocytes [3], epithelial cells [4] and tumor cells [5,6,7]. Exosomes have also been found present in physiological and pathological fluids, including plasma, urine, saliva, breast milk, malignant effusions and bronchoalveolar lavage fluid [8,9,10,11]. Exosomes mediate the disposal of obsolete membranes from original cells [12], aid in intercellular communication, modulate selected cellular activities [13] or modify their growth and mobility capacities [14] and stimulate the proliferation, survival and adhesion of target cells [15]. However, the biological functions of exosomes remain unclear. A recent study has shown that cellular gene products, such as proteins, mRNAs and microRNAs (miRNAs), are packaged inside exosomes and are delivered into recipient cells, where they exert their respective biological effects [16]. The expression of peripheral blood miRNAs from tumor cells can remain stable, because the extracellular nucleic acids in the bloodstream are highly protected by exosomes, microvesicle-like structures that can resist the degradation of various enzymes [17].

Esophageal cancer is a common type of cancer worldwide. However, esophageal cancer with distant metastasis and local invasion is still associated with a poor prognosis. To date, endoscopic and radiologic examinations are still being used as early detection methods. Simple and non-invasive diagnostic methods, such as blood and urine esophageal squamous cell carcinoma (ESCC) screening, are currently unavailable [18]. Tumor-derived exosomes may become systemic by distribution through the blood stream; hence, exosomes in the blood or other body fluids of tumor patients can be used for clinical testing [19]. The miRNA profiles of tumor-derived circulating exosomes isolated from body fluids may be used as novel diagnostic biomarkers when tumor cell materials are inaccessible. This strategy can also aid in the development of novel tumor therapeutics.

Next-generation RNA sequencing technology has rapidly progressed in recent years and is now widely used in various biological applications. This technology allows the rapid, sensitive and precise procurement of all RNA subtypes, as well as unannotated novel miRNA candidates or low-abundance RNAs that are weakly expressed [20,21,22]. On the basis of small RNA digital analysis through Solexa high-throughput sequencing, the commercially available high-throughput sequencing technology allows the identification of numerous short RNAs in a sample. miRNA sequences are 19–25 nt long, thus, the Solexa platform is apparently a suitable choice for miRNA discovery [23]. This technology can be used to explore the specific miRNA expression and distribution characteristics in tumor-derived exosomes and to discover novel miRNAs [21]. In the present study, Solexa high-throughput sequencing was used to explore the miRNA expression profile and distribution characteristics of exosomes derived from human esophageal cancer cells.

We utilized the next-generation RNA sequencing technology to construct two small-RNA cDNA libraries from human esophageal cancer cells and esophageal cancer-derived exosomes. This technology can describe the expression of miRNAs in both intracellular and extracellular environments of esophageal cancer. We identified miRNAs in the two libraries and analyzed differentially-expressed miRNAs through high-throughput sequencing and bioinformatics analysis. We validated several miRNAs and their expression profiles in esophageal cancer cells and esophageal cancer-derived exosomes through stem-loop RT-PCR. This study provided insights into the involvement of exosome-mediated miRNAs in regulating an esophageal cancer microenvironment. We identified and profiled the RNA species present in exosomes and original cells; our results suggested that exosomal miRNAs can be used as potential esophageal cancer-specific biomarkers.

## 2. Results

### 2.1. Characterization of Exosomes Released by Esophageal Cancer Cells

**Figure 1:**
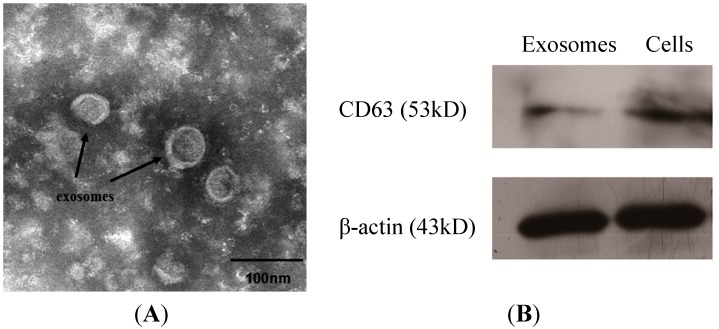
Characterization of exosomes through transmission electron microscopy and Western blot. (**A**) Exosomes isolated from the culture supernatant of EC9706 cells have a saucer-like shape that was limited by a lipid bilayer and diameters ranging from ~30–60 nm. Black arrowheads point to exosomes. Scale bar = 100 nm; (**B**) Both exosomes and cells are positive for *CD63*, as determined through Western blot analysis. *β-actin* was used as a positive control.

Based on the unique size and density of exosomes, we isolated exosomes from the culture supernatant of EC9706 cells following a classical ultracentrifugation protocol. Figure 1A shows that the purified small vesicles had diameters ranging from approximately 30–60 nm and a lipid bilayer. Western blot analysis confirmed the presence of the known exosomal membrane protein, *CD63*, which is an exosomal marker; in addition, the cells tested positive for *CD63* and *β-actin* (Figure 1B).

### 2.2. Overview of Small RNA Sequencing Data

**Figure 2:**
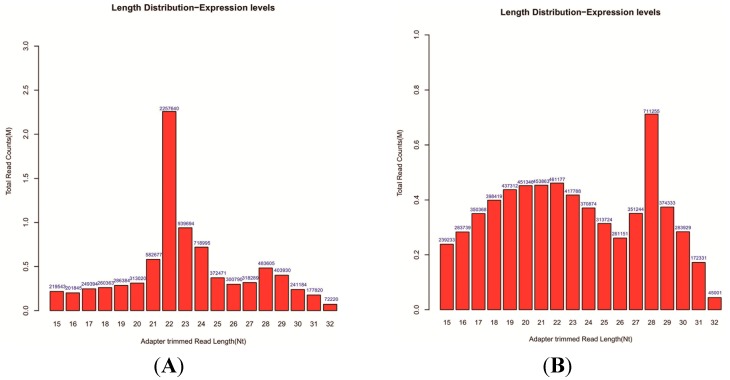
Length distribution of small RNAs in (**A**) cells and (**B**) exosomes.

To increase the coverage of cellular and exosomal miRNAs through Solexa sequencing, two small RNA libraries were constructed from cell and exosome RNA samples collected from the human esophageal cancer cell line, EC9706. After removing low-quality reads, contaminants, adaptors and sequence reads (length < 15 nt), we obtained 13,088,424 clean reads that represent 1,919,950 unique small RNAs in the cells and 7,736,476 clean reads that represent 1,226,905 unique small RNAs in the cotyledon. To simplify the sequencing data, all identical sequence reads in the small RNA library were grouped and converted into sequence tags. In total, 9,595,761 (cells) and 7,193,132 (exosomes) unique small RNAs with sizes ranging from 15–32 nt were detected from the two constructed libraries. To guarantee the accuracy of sequence data, only the sequences that were observed more than twice were selected as small RNA molecules (Table 1). Further analysis indicated that several reads were annotated, including miRNA, mRNA, rRNA, tRNA, snRNA, other small RNAs and genomic repeats. The number of miRNAs was significantly small; however, the most abundant class of small RNAs in the cells and exosomes was unannotated small RNA (Table 1). This finding indicated that a significant number of unannotated small RNAs that regulate gene expression in EC9706 cells and exosomes were secreted by the cells. The unannotated reads were used to identify novel miRNAs in subsequent analyses. The length distribution of unique small RNA sequences (15–32 nt) in the cells and exosomes remarkably varied. The lengths of unique small RNAs in the cells ranged from 21–24 nt. The most abundant size class was 22 nt, followed by 23 nt and then by 24 nt. Moreover, the lengths of unique small RNAs in the exosomes ranged from 19–22 nt; the most abundant size class was 28 nt (Figure 2). This result can be attributed to the fact that the isomiRs (miRNA mature variants) in the exosomes were more clearly identified than the other small RNAs. The above phenomenon illustrates that the size class is miRNA-focused in the small RNAs and ranges from 15–32 nt in both cells and exosomes.

**Table 1:** Summary of high-throughput sequencing data detected in cells and exosomes.

Class of Small RNAs	Cells		Exosomes	
	Number of Unique Reads	Total Number of Reads	Number of Unique Reads	Total Number of Reads
Clean Reads		13,088,424		7,736,476
Adaptor-Trimmed Reads (≥15 bp)	1,919,950	9,595,761	1,226,905	7,193,132
Protein-Coding mRNA	59,655 (3.11%)	92,372 (0.96%)	5988 (0.49%)	21,742 (0.30%)
Repbase	31,421 (1.64%)	106,725 (1.11%)	5066 (0.41%)	14,454 (0.20%)
miRNA	56,421 (2.94%)	3,163,192 (32.96%)	7726 (0.63%)	94,141 (1.31%)
rRNA	260,307 (13.56%)	1,423,180 (14.83%)	85,303 (6.95%)	542,830 (7.55%)
tRNA	80,635 (4.20%)	856,084 (9.02%)	119,500 (9.74%)	2,325,394 (32.33%)
snRNA	27,935 (1.45%)	297,457 (3.10%)	1823 (0.15%)	5472 (0.08%)
Unannotated Reads	1,403,502 (73.10%)	3,647,636 (38.01%)	1,001,224 (81.61%)	4,188,375 (58.23%)

### 2.3. Identification of Known miRNAs

**Figure 3:**
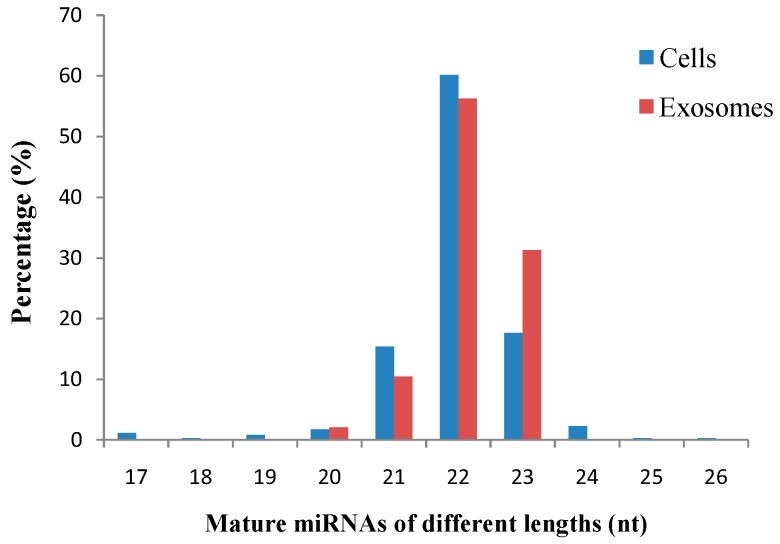
Length distribution of mature miRNAs in cells and exosomes compared with known human miRNAs in miRBase 18.0.

**Figure 4:**
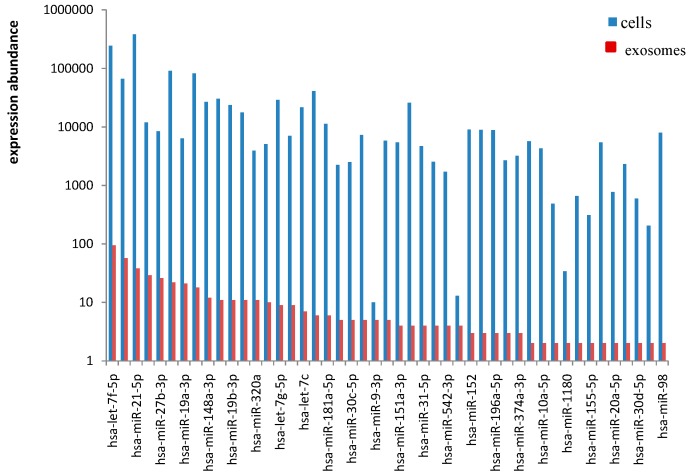
Expression abundance of known miRNA families in cells and exosomes.

**Figure 5:**
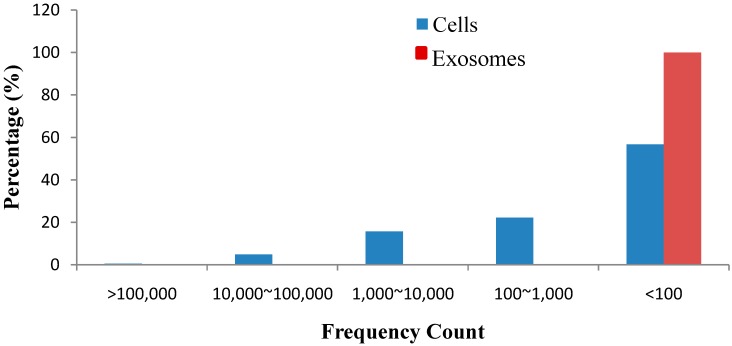
Overall expression levels of known miRNAs in cells and exosomes.

To identify the conserved miRNAs, all unique small RNA clean reads from the small RNA libraries of the cells and exosomes were compared with the known human miRNAs in miRBase 18.0 (University of Manchester, Manchester, UK). We identified 342 and 48 types of published mature miRNAs in cells and exosomes, respectively. The lengths of the identified mature miRNAs ranged from 17–26 and 20–23 nt in cells and exosomes, respectively. Among all of the identified mature miRNAs, 22 nt-long miRNAs accounted for the highest proportions of 57.91% (cells) and 56.06% (exosomes) (Figure 3). The absolute sequence reads were transformed into transcript abundance by data normalization. The clean read counts of miRNAs can represent the corresponding expression levels of each set of miRNAs. The miRNA reads varied from two to 382,634, indicating that the reads can still be detected through Solexa high-throughput, sequencing regardless of the miRNA expression level. Accordingly, miRNAs, such as *mir-21-5p* and *let-7f-5p*, were highly expressed, whereas *mir-9-3p* and *mir-9-5p* were lowly expressed (Figure 4). The clean read counts ranged from <10 to >100,000, which is across five orders of magnitude (Figure 5).

### 2.4. EC9706 and Their Corresponding Exosomes Contain a Subset of Dysregulated miRNA

**Figure 6:**
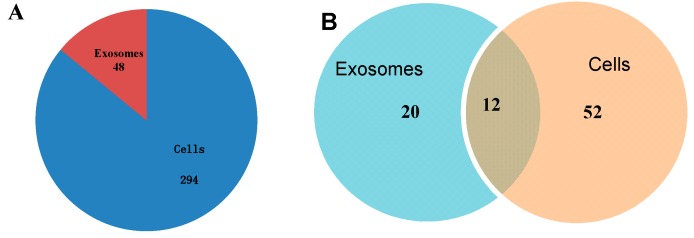
Profiling of miRNAs isolated from EC9706 and their corresponding exosomes. (**A**) Pie chart of detected known miRNAs in cells and exosomes; and (**B**) Venn diagram of detected novel miRNAs in cells and exosomes.

We summarized the common and specific sequences between the cells and exosomes. Utilizing the isolated miRNA from EC9706 and their corresponding exosomes, we profiled the miRNAs and found 342 known miRNAs in the cells, 48 of which were detected in the exosomes. Figure 6 shows the number of shared and specific miRNAs between the EC9706-derived exosomes and cells. The cells contained 294 miRNAs that were not detected in the exosomes. Meanwhile, 64 and 32 novel miRNAs were detected in the cells and exosomes, respectively. Moreover, 12 of these novel miRNAs were detected in the two samples. Remarkably, several high-number miRNAs were selectively identified in the exosomes, but not in the cells. This result may be attributed to certain miRNAs that were transferred into the exosomes at low levels within the cells. This phenomenon may result in missing miRNAs when the cDNA library in the course of human miRBase establishment was constructed. Table 2 and Table 3 show the top-ranking miRNA transcripts in the intracellular and extracellular spaces, respectively. Noticeably, the *let-7* family of miRNAs is predominantly represented in the top-ranking miRNAs in both intracellular and extracellular samples of esophageal cancer. In addition to the *let-7* family, other miRNAs, such as *miR-21-5p* and *miR-26a-5p*, were also highly expressed in the intracellular and extracellular esophageal cancer samples. All known miRNAs in exosomes were detected at lower levels compared with their corresponding cells, whereas several novel miRNAs in exosomes were detected at higher levels compared with their corresponding cells (Table 3 and Table 4). Among the 12 common novel miRNAs, eight miRNAs were upregulated by more than two-fold in the exosomes, and four miRNAs were down-regulated by less than two-fold in the cells. Overall, these results show that the miRNA profiles in the cells and exosomes are notably different, which agree with the observations that miRNA is sorted and released in exosomes through unknown mechanisms [24].

**Table 2:** Common transcripts in intracellular samples in the high category of more than 10,000 transcripts.

miRNA Annotation	Pre-miRNA Arm (5p or 3p)	Mature MicroRNA Seed	Transcript Sequence	Is miRBase Mature miRNA the Most Abundant Sequence?	Intracellular Transcript Number
hsa-miR-21-5p	5p	AGCTTA	TAGCTTATCAGACTGATGTTGA	Yes	382,634
hsa-let-7f-5p	5p	GAGGTA	TGAGGTAGTAGATTGTATAGTT	Yes	243,882
hsa-let-7b-5p	5p	GAGGTA	TGAGGTAGTAGGTTGTGTGGTT	Yes	91,479
hsa-miR-100-5p	5p	ACCCGT	AACCCGTAGATCCGAACTTGTG	Yes	82,325
hsa-let-7a-5p	5p	GAGGTA	TGAGGTAGTAGGTTGTATAGTT	Yes	66,589
hsa-miR-125b-5p	5p	CCCTGA	TCCCTGAGACCCTAACTTGTGA	Yes	41,096
hsa-let-7i-5p	5p	GAGGTA	TGAGGTAGTAGTTTGTGCTGTT	Yes	30,233
hsa-let-7g-5p	5p	GAGGTA	TGAGGTAGTAGTTTGTACAGTT	Yes	28,900
hsa-miR-148a-3p	3p	CAGTGC	TCAGTGCACTACAGAACTTTGT	Yes	26,923
hsa-miR-24-3p	3p	GGCTCA	TGGCTCAGTTCAGCAGGAACAG	Yes	26,085
hsa-miR-19b-3p	3p	GTGCAA	TGTGCAAATCCATGCAAAACTGA	Yes	23,649
hsa-let-7c	5p	GAGGTA	TGAGGTAGTAGGTTGTATGGTT	Yes	21,557
hsa-miR-25-3p	3p	ATTGCA	CATTGCACTTGTCTCGGTCTGA	Yes	17,757
hsa-miR-182-5p	5p	TTGGCA	TTTGGCAATGGTAGAACTCACACT	Yes	15,213
hsa-miR-425-5p	5p	ATGACA	AATGACACGATCACTCCCGTTGA	No	12,236
hsa-miR-26a-5p	5p	TCAAGT	TTCAAGTAATCCAGGATAGGCT	Yes	11,993
hsa-miR-181a-5p	5p	ACATTC	AACATTCAACGCTGTCGGTGAGT	Yes	11,329
hsa-miR-99a-5p	5p	ACCCGT	AACCCGTAGATCCGATCTTGTG	Yes	10,476
hsa-miR-103a-3p	3p	GCAGCA	AGCAGCATTGTACAGGGCTATGA	Yes	10,305

**Table 3:** Common transcripts in extracellular samples that belong to the mid-range category with five to 100 transcripts. No transcripts with counts higher than 100 were observed in the extracellular space.

miRNA Annotation		Pre-miRNA Arm (5p or 3p)	Mature MicroRNA Seed	Transcript Sequence	Is miRBase Mature miRNA the Most Abundant Sequence?	Extracellular Transcript Number
hsa-let-7f-5p		5p	GAGGTA	TGAGGTAGTAGATTGTATAGTT	Yes	95
hsa-let-7a-5p		5p	GAGGTA	TGAGGTAGTAGGTTGTATAGTT	Yes	57
hsa-miR-21-5p		5p	AGCTTA	TAGCTTATCAGACTGATGTTGA	Yes	38
hsa-miR-26a-5p	5p		TCAAGT	TTCAAGTAATCCAGGATAGGCT	Yes	29
hsa-miR-27b-3p	3p		TCACAG	TTCACAGTGGCTAAGTTCTGC	Yes	26
hsa-let-7b-5p	5p		GAGGTA	TGAGGTAGTAGGTTGTGTGGTT	Yes	22
hsa-miR-19a-3p	3p		GTGCAA	TGTGCAAATCTATGCAAAACTGA	Yes	21
hsa-miR-100-5p	5p		ACCCGT	AACCCGTAGATCCGAACTTGTG	Yes	18
hsa-miR-148a-3p	3p		CAGTGC	TCAGTGCACTACAGAACTTTGT	Yes	12
hsa-let-7i-5p	5p		GAGGTA	TGAGGTAGTAGTTTGTGCTGTT	Yes	11
hsa-miR-19b-3p	3p		GTGCAA	TGTGCAAATCCATGCAAAACTGA	Yes	11
hsa-miR-25-3p	3p		ATTGCA	CATTGCACTTGTCTCGGTCTGA	Yes	11
hsa-miR-320a	3p		AAAGCT	AAAAGCTGGGTTGAGAGGGCGA	Yes	11
hsa-miR-423-5p	5p		GAGGGG	TGAGGGGCAGAGAGCGAGACTTT	Yes	10
hsa-let-7g-5p	5p		GAGGTA	TGAGGTAGTAGTTTGTACAGTT	Yes	9
hsa-miR-92a-3p	3p		ATTGCA	TATTGCACTTGTCCCGGCCTGT	Yes	9
hsa-let-7c	5p		GAGGTA	TGAGGTAGTAGGTTGTATGGTT	Yes	7
hsa-miR-125b-5p	5p		CCCTGA	TCCCTGAGACCCTAACTTGTGA	Yes	6
hsa-miR-181a-5p	5p		ACATTC	AACATTCAACGCTGTCGGTGAGT	Yes	6

**Table 4:** Top 10 novel miRNAs expressed in exosome libraries.

Mature MicroRNA ID	Pre-miRNA Arm (5p or 3p)	Mature MicroRNA Seed	Most Abundant Sequence (isomiR)	Is miRBase Mature miRNA the Most Abundant Sequence?	Cell Counts	Exosome Counts
hsa-miR-chr12_3064	3p	GAGAGG	GGAGAGGTGGATGAGTGGTTTA	No	0	339
hsa-miR-chr1_9487	3p	GAGAGG	GGAGAGGTGGATGAGTGGTTTA	No	0	339
hsa-miR-chr15_4885	3p	TTCAAG	GTTCAAGTCCAGCTGGG	Yes	30	306
hsa-miR-chr3_14243	3p	TTCAAG	GTTCAAGTCCAGCTGGG	Yes	30	306
hsa-miR-chr18_7463	3p	ACGTGA	CACGTGAAACCCTGTCTGAAT	No	14	137
hsa-miR-chr2_11998	5p	AGGACT	AAGGCAGGACTGGTGACTGGGGTG	No	7	59
hsa-miR-chrY_24624	3p	TCCCTG	GTGTCCCTGGTTCGAGCCC	No	0	55
hsa-miR-chr6_18451	3p	GTCGTG	GGTCGTGGGTTCGAGC	No	0	37
hsa-miR-chr20_11216	3p	GTTCGA	GGTTCAAATCCTGTCTTCT	No	2	28
hsa-miR-chr1_9933	5p	TCTTTG	ATCTCTTTGAGTTCTCACCA	No	2	19

### 2.5. Prediction of Novel miRNAs

To identify potential novel miRNAs from the small RNA library of EC9706 cells and exosomes, the following approaches and criteria were used: (i) reads that matched known miRNAs (miRBase 18.0) and other non-coding RNAs were excluded; (ii) non-conserved unique reads that were only sequenced once were removed; and (iii) to be considered as new miRNAs, reads must be entirely within the arm of the hairpin, and the hairpin must not possess large internal loops and bulges [25]. Based on the criteria for miRNAs used in the present study, we detected 64 and 32 novel miRNA candidates in cells and exosomes, respectively, using miRDeep2 software [26]. The 12 miRNAs found in both libraries can be divided into two categories, namely, miRNAs with only a single locus and those with multiple loci (Table 5 and Table 6). The expression levels of the candidate miRNAs in the cells and exosomes were significantly different. In addition, the length of these newly identified miRNA sequences varied from 17–25 nt, in which the 17, 18 and 22 nt miRNAs were dominant.

**Table 5:** Candidate miRNAs with only a single locus.

Candidate miRNA ID	Precursor Coordinate	Consensus Mature Sequence	Length	Pre-miRNA Arm (5p or 3p)	Cell Counts	Exosome Counts
hsa-miR-chr18_7463	chr18:42911426..42911487: +	CACGTGAAACCCTGTCT	17	3p	14	137
hsa-miR-chr2_11998	chr2:9376082..9376166: +	CAGGACTGGGGACTGGGGTG	20	5p	7	59
hsa-miR-chr20_11216	chr20:39392006..39392060: −	GGTTCGAATCCTGTCTTCT	19	3p	2	28
hsa-miR-chr1_9933	chr1:45874140..45874224: −	CTCTTTGAGTTCTCACCA	18	5p	2	19
hsa-miR-chr5_17564	chr5:33712846..33712885: −	GTACTCAAGAGGCTGAAGA	19	5p	7	18
hsa-miR-chr1_8755	chr1:62567393..62567462: +	TCAAATCCTGTCTGACC	17	3p	2	17
hsa-miR-chr7_20613	chr7:25989517..25989563: −	TCAGTGCACTACAGAACTTTGT	22	5p	26,923	12
hsa-miR-chr11_1761	chr11:31858407..31858458: −	GCATGGGTGGTTCAGTGGTAGAATT	25	5p	202	5
hsa-miR-chr19_8205	chr19:13947044..13947124: −	TGGCTCAGTTCAGCAGGAACAG	22	5p	26,085	4
hsa-miR-chr16_5626	chr16:14397874..14397964: +	AACTGGCCCTCAAAGTCCCGCT	22	5p	5460	2

+, positive-sense strand in genomic DNA; −, antisense strand in genomic DNA.

**Table 6:** Candidate miRNAs with multiple loci.

Candidate miRNA ID	Precursor Coordinate	Consensus Mature Sequence	Length	Pre-miRNA Arm (5p or 3p)	Cell Counts	Exosome Counts
hsa-miR-chr15_4885	chr15:43193729..43193791: +	GTTCAAGTCCAGCTGGG	17	3p	30	306
hsa-miR-chr3_14243	chr3:101824879..101824941: +	GTTCAAGTCCAGCTGGG	17	3p	30	306

+, positive-sense strand in genomic DNA.

### 2.6. Sequence Variations in miRNAs

**Figure 7:**
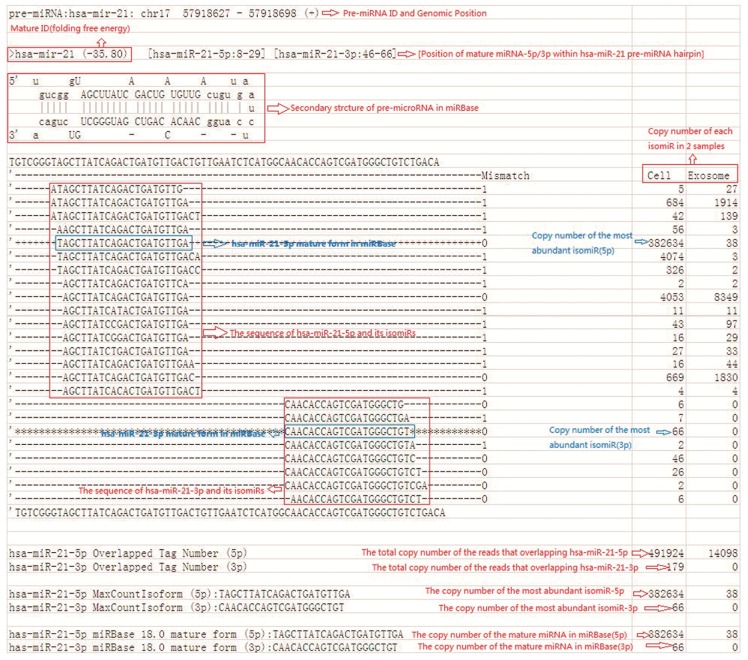
Example of *has-miR-21* annotation for sample cells and exosomes. Various isomiRs are observed for several known miRNAs. The reference miRNA sequence from miRBase is, in a few cases, not the most frequently observed isomiR. The repertoire of *has-miR-21* and its isomiRs contains the following: structure and sequence information of *has-miR-21* pre-miRNA in miRBase, including ID, genomic location, folding free energy, and its secondary structure; sequence and read counts of all isomiRs aligned to *has-miR-21* pre-miRNA hairpin; sequence and read counts of mature *has-miR-21* annotated in miRBase; sequence and read counts of *has-miR-21* as the most abundant isomiR; and total read counts found at the 5p or 3p arm of *has-miR-21* pre-miRNA. “+” means positive sense strand in genomic DNA.

IsomiRs are miRNA variants commonly reported in next-generation RNA sequencing studies. IsomiRs are encoded by the same pre-miRNAs and exhibit sequence variations from the reference miRNAs in miRBase [27]. Such a phenomenon was also observed in the present study. The results of Solexa sequencing revealed that most of the identified miRNAs showed length and sequence heterogeneity. Multiple unique reads that map the same position of a hairpin sequence (isomiRs) were further represented by the sequence of the most abundant read, because the read counts of the most abundant isomiR, rather than of the miRBase reference sequences, provided the most robust approach for comparing the expression levels between samples (Figure 7). In 158 cases, the most abundant sequence did not exactly correspond to the current human miRBase 18.0 reference sequences. For instance, the size of *has-miR-7* is 24 (the read number is 30,883) in our library, but 23 (the read number is 8329) in miRBase 18.0. The size of another miRNA *has-miR-222* is 23 (the read number is 16,121) in our library but 21 (the read number is 1047) in miRBase 18.0. This difference also suggests that either the relative abundance of isomiRs may vary across different studies or that the original submission of this miRNA to miRBase is incorrect [28].

### 2.7. miRNA Validation Assays by qRT-PCR

**Figure 8:**
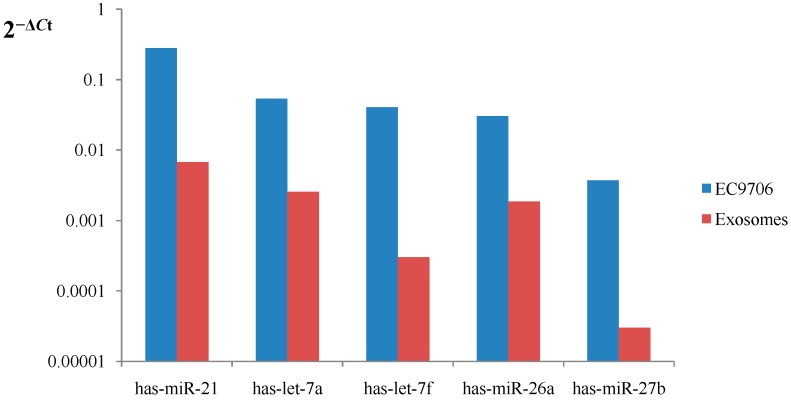
Expression profiles of five miRNAs in cells and exosomes. Total RNA isolated from cells and exosomes were analyzed through qRT-PCR. ∆*C*_t_ values normalized against *RNU6B* (cells) and *cel-miR-39* (exosomes) levels were calculated, representing the relative expression levels of five target miRNAs.

To determine whether or not the changes that occurred within exosomes also occurred in cells, qRT-PCR analysis of miRNA was performed on EC9706 and exosomes released from the cells. In agreement with the sequence data, this analysis confirmed that the expression levels of the five miRNAs that showed the highest expression in exosomes were relatively similar to those in cells (Figure 8).

## 3. Discussion

miRNAs are important members of small, well-conserved and non-coding RNAs. Their function in cancer pathogenesis as oncogenes or tumor suppressors has been investigated and attracted attention for several years [29]. With the development of next-generation RNA sequencing technology, an increasing number of miRNAs in tumor-derived exosomes has been discovered [22,30]. However, the roles of miRNAs in esophageal cancer-derived exosomes remain unknown. Serum *miR-18a* level was significantly higher in patients with esophageal, pancreatic or hepatocellular cancer. The areas under the receiver-operating characteristic curve (ROC) were 0.944, 0.936 and 0.881 in esophageal, pancreatic and hepatocellular, respectively, which showed that serum *miR-18a* may serve as a screening biomarker [31]. Researchers have characterized the miRNA profiles of colorectal cancer (CRC) serum exosomes using microarray analyses to identify several special miRNAs up-regulated in CRC patients and found that the levels of seven miRNAs are significantly down-regulated after surgical resection of tumors. Through ROC analysis, *miR-23a* and *miR-1246* were detected to have higher sensitivities of 95% and 90%, respectively, for Stage I samples compared with known tumor markers (CA19-9 and CEA). This finding supports the promising application of *miR-23a* and *miR-1246* as diagnostic biomarkers [32]. Another study on the serum microRNA expression profile of esophageal cancer showed that the expression level of *miR-1246* is higher in ESCC patients than in controls and that this expression level decreases after surgical resection. To assess the diagnostic and prognostic values of serum *miR-1246* in ESCC, the ROC curve assay of *miR-1246* was conducted. Results showed that the sensitivity and specificity of *miR-1246* were 71.3% and 73.9%, respectively, in distinguishing ESCC patients from healthy controls. The abundant exosomal circulating *miR-1246* in serum significantly correlates with the ESCC tumor-node-metastasis stage, and it is the most potent independent risk factor for poor survival [33]. Tanaka *et al.* [18] analyzed the exosomes in serum from patients who suffered from ESCC and confirmed that exosomal *miR-21* expression is significantly higher in the serum of ESCC patients than in that of benign controls. Exosomal *miR-21* expression is related to clinicopathological stage, tumor classification, positive lymph node status and metastatic status; therefore, *miR-21* has been suggested to be a biomarker for detecting ESCC progression. In the present study, we developed a workflow for analyzing next-generation RNA sequencing of data that focus on miRNAs in human esophageal cancer cells and their corresponding exosomes. Using this technology, we detected 9,595,761 (cells) and 7,193,132 (exosomes) unique small RNAs. This result indicates the presence of a significant number of small RNA in exosomes, among which, miRNAs predominate. This workflow has revealed several trends in known and novel miRNA expression profiles for esophageal cancer-derived exosomes. A total of 80 miRNAs, including 48 known and 32 novel miRNAs, were firstly reported in esophageal cancer cell-derived exosomes. In addition, comparison with the miRBase 18.0 revealed several common miRNAs that were expressed in the cells and exosomes. The expression levels of most known miRNAs were significantly lower in the exosomes than in the cells. We also found several novel miRNAs that presented different profiles between the cells and their corresponding exosomes. Several novel miRNAs were expressed at higher levels in the exosomes than in the cells, implying that these novel miRNAs may be uniquely packaged into exosomes with specialized, but unknown functions. This phenomenon may be also caused by the fact that most studies only focused on miRNAs with high read counts in cells. The studies based on miRNAs are also generally from high to low read counts to establish a miRNA database. Some novel miRNAs are highly expressed in exosomes, but lowly expressed in cells. Taylor *et al.* [34] studied the miRNA expression in ovarian tumor cells and their corresponding exosomes. Among the 218 mature miRNAs that were positive in both cells and exosomes, 12 and 31 were elevated in cells and exosomes, respectively. Bellingham *et al.* [35] proposed a selective mechanism for the incorporation and release of miRNAs in exosomes that contain limited or no *18S* and *28S* cellular ribosomal species. Not all mRNAs and miRNAs contained within cells can be directly targeted and packaged in exosomes. Therefore, the differential expression profiles of miRNAs in the cells and exosomes probably have different biological functions.

In the present study, *has-miR-21*, *has-let-7* family, *miR-26a* and *miR-27b* were abundant in EC9706 cells and their corresponding exosomes. Known miRNA species have been previously sorted into exosomes in other cell lines. Chiba *et al.* [36] revealed that exosomes derived from the colorectal cancer cell lines contain miRNAs, such as *miR-21*, and can be delivered into recipient cells through exosomes. Moreover, studies on *miR-21* and esophageal cancer revealed that *miR-21* is post-transcriptionally regulated by phosphatase and tensin (*PTEN*) via binding to the 3'-UTR of *PTEN* mRNA. Consequently, *PTEN* inhibits tumor cell growth and invasion by blocking the PI3K/AKT pathway [37,38]. Yan *et al.* [39] found, through locked nucleic acid silencing combined with microarray technology, that *miR-21* knockdown can inhibit the growth and migration of breast cells *in vitro* and tumor growth in nude mice. A previous study found that the *let-7* miRNA family is abundant in both the intracellular and extracellular fractions of a metastatic gastric cancer cell line (AZ-P7a); this study suggested that AZ-P7a cells release *let-7* miRNAs via exosomes into the extracellular environment to maintain their oncogenesis [16]. Kobayashi *et al.* [40] determined that the miRNA transcripts of the *let-7* family exist in both ovarian cancer cell lines and their exosomes; they established that the release and miRNA content of exosomes significantly differ between ovarian cancer cell lines and correlate with their invasive potential. Colamaio *et al.* [41] demonstrated that the *let-7a* overexpression in the follicular thyroid carcinoma cells increases cell adhesion and reduces cell migration; however, *let-7a* silencing in normal rat thyroid cells induces the opposite effects through stable transfections. *Has-miR-26a* and *has-miR-27b* were both found in ovarian tumor cells and their corresponding exosomes; however, their expression levels significantly differ between the two samples [34]. Several studies on breast cancer have shown that *miR-26a* can inhibit cell proliferation, colony formation and migration, as well as promote apoptosis by regulating several carcinogenesis-related processes, including several mechanisms that involve the targeting of *MCL-1*, *MTDH* and *EZH2* [42,43]. The altered expression levels of different miRNAs involved in multiple signaling transduction pathways can determine cancer development and/or progression by interacting with one another. Namwat *et al.* [44] showed that the oncogenic factor *miR-21* is up-regulated, while the tumor suppressor *let-7a* is down-regulated in human cholangiocarcinoma tissues. The high level of IL-6 in such a chronic inflammation-related cancer possibly stimulates *miR-21* expression, and *RAS* activation is partly correlated with *let-7a* down-regulation. Kida *et al.* [45] found that the *PPARα* protein level in the human hepatocellular carcinoma cell line HuH7 significantly decreases when the levels of *miR-21* or *miR-27b* are over-expressed and inhibited, respectively. This result suggests that the two miRNAs may serve as important regulators for the fatty acid catabolism of liver cells. Another study on miRNAs in oral squamous cell carcinoma (OSCC) reported that increasing *miR-125b* and *miR-100* levels reduces cell proliferation; however, co-transfecting the two miRNAs significantly influences proliferation than transfecting OSCC cells individually. Researchers proposed that the reduced proliferation observed in cells co-transfected with *miR-125b* and *miR-100* is caused by the additive effect of the two miRNAs on gene expression rather than a synergistic change [46]. miRNA expression profiles were previously confined to tissues and cells; today, circulating miRNAs reportedly exist in the peripheral blood with exosomes as carriers. The effective delivery of miRNAs via exosomes in the bloodstream has provided a new mechanism for cell-to-cell communication. Investigating the mechanisms by which these candidate miRNAs carried by exosomes function in esophageal cancer development is important.

Our study revealed a significant number of isomiRs derived from almost all detected miRNAs. In total, 17,111 known and 5212 novel isomiRs were detected in our two libraries. Most isomiRs showed variability at their 5' and/or 3' ends, likely resulting from the variability in either Dicer or Drosha cleavage positions within the pre-miRNA hairpin; an example of which is given in Figure 8, which shows sequenced isomiRs for *miR-21* with high read counts in cells and exosomes. This result indicates that the majority of esophageal cancer-derived miRNA nucleotide variants resulted from post-transcriptional modifications. IsomiRs have several biological functions, such as the amelioration of the signal-to-noise ratio in miRNA–mRNA communication by targeting miRNA-controlled genetic networks. A close correlation exists between the expression characteristics of isomiRs and canonical miRNAs. These isomiRs are functional, and their biological roles likely involve the improvement of the signal-to-noise ratio in miRNA–mRNA communication by targeting miRNA-controlled genetic networks [47]. This finding suggests that isomiRs may have various functions. However, further studies need to be conducted for verification.

Recent studies have suggested that tumor-derived exosomes present a novel mechanism for intercellular communication [48,49]. The miRNA profiles of circulating exosomes from the body fluids of patients with tumors have also been proposed to be used as signatures for disease diagnosis. Furthermore, small non-coding RNA signatures exist in microvesicles isolated from glioblastoma multiforme (GBM) patient serum. A study has shown that exosomal miRNA expression levels are significantly higher in 25 patients than in age- and sex-matched healthy controls [50]. A study on pancreatic adenocarcinoma (PC) revealed that serum exosomal *miR-17-5p* and *miR-21* levels are significantly higher in PC patients than in healthy participants and non-primary carcinoma patients; this result suggests that serum exosomal miRNAs may serve as potential biomarkers of PC [51]. Furthermore, a study on exosomal microRNA in lung cancer indicated that a significant difference in total exosome and exosomal miRNA levels exists between lung cancer patients and controls; this study also noted that exosomal miRNA profiling has the potential to be used as a screening tool for cancer detection because of its minimal invasiveness [52].

This study is the first to report that exosomes released from esophageal cancer cells contain a series of miRNAs and that the profiles of several exosomal miRNAs resemble those of their parent cells. However, several novel miRNAs in exosomes still need to be investigated. In conclusion, the discovery of these known and novel miRNAs using Solexa high-throughput sequencing provided new insights into the expression profiles and distribution characteristics of miRNAs in esophageal cancer-derived exosomes and afforded significant values to further explore miRNAs in intercellular communication mediated by exosomes in esophageal cancer.

## 4. Experimental Section

### 4.1. Cell Culture

The human esophageal cancer cell line EC9706 was purchased from Shanghai Tiancheng Science and Technology Co., Ltd. EC9706 cells were cultured in RPMI-1640 medium (Hyclone, Logan, UT, USA) supplemented with 10% heat-inactivated fetal bovine serum (Hyclone), 100 U/mL penicillin and 100 U/mL streptomycin under a humidified 5% CO_2_ atmosphere at 37 °C. The cells were centrifuged at 10,000× *g* for 30 min to remove exosomes in fetal bovine serum and then ultracentrifuged (Beckman Coulter, Brea, CA, USA) at 200,000× *g* for 6 h to remove bovine-derived exosomes.

### 4.2. Exosome Isolation

EC9706 cells (40 mL) at a density of 1 × 10^8^ cells per 175-cm^2^ flask were cultured in complete RPMI-1640 medium at 37 °C and 5% CO_2_. After 48 h, exosomes were isolated and purified from the culture medium of EC9706 using a sequential centrifugation protocol. Briefly, the culture medium was collected and centrifuged at 300× *g* for 10 min, 800× *g* for 10 min, 1200× *g* for 20 min and 10,000× *g* for 30 min to remove lifted cells and cellular debris. The supernatant was then ultracentrifuged at 100,000× *g* for 3 h using a 70Ti rotor (Beckman Coulter) to pelletize the exosomes. The exosome pellets were washed with filtered phosphate-buffered saline (PBS) and then recentrifuged at 100,000× *g* for 2 h. The supernatant was removed, and the final exosomal pellet was resuspended in 100 μL of PBS. All centrifugation steps were conducted at 4 °C.

### 4.3. Transmission Electron Microscopy

A 20-μL aliquot of the suspension was loaded onto a carbon-coated grid for 2 min at room temperature. The grid was positioned with the coating side facing the drop containing exosomes. The samples were fixed by covering the grid with 10 μL of 1% aqueous phosphotungstic acid for 1 min and then observed under a transmission electron microscope (Hitachi, Shiga, Japan).

### 4.4. Western Blot

Samples of cells and exosomes were washed and resuspended in RIPA lysis buffer (Bi Yuntian, Hefei, China) with protease inhibitor mixture. After centrifuging at 14,000× *g* and for 5 min at 4 °C, the supernatant was collected, and the protein concentration was determined using a BCA Protein Assay Kit (Generay, Shanghai, China). Up to 67 μg of proteins were denatured by boiling in sodium dodecyl sulfate (SDS) loading buffer, loaded onto SDS-polyacrylamide gels and then transferred onto polyvinylidene difluoride membranes (0.45 μm pore size, Millipore, Billerica, MA, USA). The blots were blocked overnight with 5% non-fat dry milk in Tris-buffered saline containing Tween 20. After blocking, the membranes were incubated with primary mouse monoclonal antibody anti-CD63 (1:1000) (sc-51662, Santa Cruz, CA, USA) or mouse anti-β-actin (1:400, internal standard) (BM0627, Boster, Wuhan, China) in TBST for 1.5 h at room temperature. The membranes were washed six times for 5 min each before incubating with the secondary antibody. The secondary antibody conjugated with horseradish peroxidase was incubated at a dilution ratio of 1:5000 for 1 h at room temperature. Immunoreactive bands were visualized using enhanced chemiluminescence (Thermo, Pittsburgh, PA, USA) and Image Scanner III (EPSON, Tokyo, Japan).

### 4.5. RNA Isolation and Analysis

Total RNAs from exosomes and cultured cells were isolated using the mirVana miRNA Isolation Kit (Ambion, Austin, TX, USA) according to the manufacturer’s total RNA isolation procedure. The RNA quality and concentration were assessed with a 260/280 ratio using a NanoDrop spectrophotometer (NanoDrop ND-1000 Technologies, Inc., Wilmington, DE, USA).

### 4.6. Small RNA Libraries Construction and Solexa Sequencing

Approximately 1 μg of total RNA from each sample was used to prepare the miRNA sequencing library. The 3'-adapter was ligated to the isolated small RNAs using T4 RNA ligase (Epicentre, San Diego, CA, USA). The 5'-adapter was ligated to the isolated small RNAs using T4 RNA ligase. The adapter-ligated sRNAs were used as templates for RT-PCR to amplify single-stranded cDNA templates to double-stranded cDNAs. Then, ~110–130-bp PCR-amplified fragments (correspond to ~15–32 nt small RNAs) from the 15% denaturation PAGE gels were extracted and then purified. After the completed libraries were quantified using Agilent 2100 Bioanalyzer (Agilent Technologies Inc., Santa Clara, CA, USA), the DNA fragments in the libraries were denatured with 0.1 M NaOH to generate single-stranded DNA molecules, captured using Illumina flow cells, amplified *in situ* and then finally sequenced for 36 cycles using a Genome Analyzer IIx (Illumina, San Diego, CA, USA) according to the manufacturer’s instructions.

### 4.7. Bioinformation Analysis of Small RNA Data

After generating the sequence reads from the Genome Analyzer IIx, image analysis and base calling were performed using Off-Line Basecaller software (OLB V1.8.0, Illumina, San Diego, CA, USA). Subsequently, 3' adapter sequences were trimmed from clean reads (reads that passed Solexa CHASTITY quality filter), and reads shorter than 15 nt were discarded. After removing the vector sequences, the modified sequences from 15–32 nt were counted and used for further analysis. The length distribution and common/specific clean reads of cells and exosomes were then analyzed. In addition, all unique sequences were used to search the ncRNA data Rfam, (European Molecular Biology Laboratory, Heidelberg, Germany) for the removal of non-miRNA sequences (rRNA, tRNA and snRNA). Clean reads that matched these sequences were excluded from further analysis. Subsequently, the unique small RNA sequences were used to perform a BLASTN search against the miRNA database miRBase 18.0 and to identify the conserved miRNAs in cells and exosomes. The unannotated clean reads were used to predict novel miRNAs and to analyze the single nucleotide variants of known miRNAs.

### 4.8. Expression Patterns of Known and Novel miRNAs

The clean reads were analyzed against the Rfam database using BLAST. The count of clean reads that originated from each miRNA can indicate the expression level of the corresponding miRNA. To understand the differential expression of conserved and novel miRNAs between cells and exosomes, the frequency of each miRNA was normalized to the same order of magnitude using the formula: normalized expression = actual miRNA count/total count of clean reads × 1,000,000 [20]. If the normalized expression level of an miRNA had read counts <2, this miRNA was removed from future differential expression analyses. Only the perfectly matched sequences were considered to be conserved miRNAs. Furthermore, the prediction of novel miRNAs of cells and exosomes was conducted using miRDeep 2 software.

### 4.9. miRNA Variants

**Figure 9:**
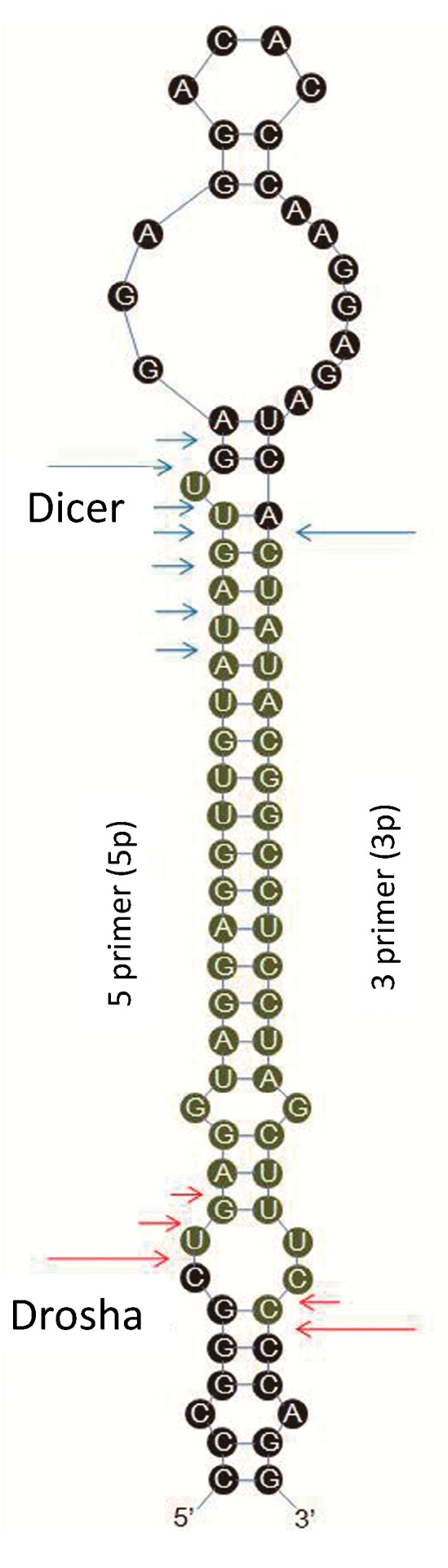
The predicted structure of pre-miRNA is presented as a graph. The experimentally inferred Drosha and Dicer cleavage positions are indicated with red and blue arrows, respectively. Long arrows represent cleavage sites for the most abundant isomiR, and short arrows indicate other isomiRs.

IsomiRs are mature variants of miRNAs from their miRBase reference sequences. The Solexa sequencing results in the present study revealed that most of the identified miRNAs had length and sequence heterogeneity. The considerable degree of isomiR variability can be attributed to the variability in either Dicer or Drosha cleavage positions within the pre-miRNA hairpin (Figure 9). To characterize the isomiR variability, sequences that matched the miRNA precursors in the mature miRNA region ±4 nt (not more than one mismatch) were considered as mature miRNA isomiRs and were grouped according to the 5' (5p) or 3' (3p) arm of the precursor hairpin.

### 4.10. Measurement of miRNA Expression Level by qRT-PCR

Total RNA was isolated from exosomes and cultured cells using a mirVana miRNA Isolation Kit (Ambion, Austin, TX, USA). qRT-PCR was performed using SYBR assay according to the manufacturer’s instructions. Each reaction mixture comprised 1 μL of RT-primer, 2.5 μL of 5× reverse-transcription buffer, 1 μL of 2.5 mM dNTPs, 0.25 μL of RNase-Inhibitor, 0.25 μL of MMLV reverse transcriptase (Promega, Madison, WI, USA) and 5 ng of RNA (exosomes) or 500 ng of RNA (cells). ddH_2_O was added to a mixed volume of 12.5 μL. The RT reactions were conducted under the following conditions: 42 °C for 60 min, 70 °C for 10 min and then held at 4 °C. For the exosomes, the total RNA content was extremely low. The RT product was pre-amplified prior to the RT-PCR step to enhance sensitivity [53]. The reaction product was then setup for RT-PCR with the ABI PRISM 7300 real-time PCR system (Applied Biosystems, Carlsbad, CA, USA). Briefly, each reaction was performed with a final volume of 10 μL containing 1 μL of DNA, 3.75 μL of SYBR Green, 0.75 μL of Plus, 1 μL of 5 µM forward primer, 1 μL of 5 μM reverse primer and 2.5 μL of ddH_2_O. The reaction mixtures were incubated at 95 °C for 5 min, followed by 40 cycles of 95 °C for 15 s, 60 °C for 30 s and 72 °C for 30 s. Fluorescence measurements were then conducted. To calculate the relative fold change values, the *C*_t_ value data were normalized to U6 and cel-miR-39 as the endogenous controls for cells and exosomes, respectively. The relative quantification (fold change) for the miRNA expression of the host cells and exosomes was determined using 2^−Δ*C*t^ (where Δ*C*_t_ = (*C*_t_ of miRNA of interest) − (*C*_t_ of endogenous control gene (U6 or cel-miR-39)). Three technological replicates were used to ensure the reliability of the quantitative analysis. The melting curves in each experiment were analyzed to examine the sensitivity and specificity of the primers.

## 5. Conclusions

This study explored the miRNA expression profile and distribution characteristics of exosomes derived from human esophageal cancer cells using the next-generation RNA sequencing technology. These findings provided new insights into the characteristics of miRNAs in exosomes derived from human esophageal cancer cells. These specific miRNAs expressed in exosomes may play important roles in intercellular communication mediated in esophageal cancer.
